# PQ, a new program for phylogeny reconstruction

**DOI:** 10.1186/s12859-018-2399-4

**Published:** 2018-10-12

**Authors:** Dmitry Penzar, Mikhail Krivozubov, Sergey Spirin

**Affiliations:** 1 Faculty of Bioengineering and Bioinformatics, Moscow State University, 1 Leninskiye Gory, bld. 73, Moscow, 119991 Russia; 2 Gamaleya Center of Epidemiology and Microbiology, 18 Gamaleya st., Moscow, 123098 Russia; 3 Belozersky Institute of Physico-Chemical Biology, Moscow State University, 1 Leninskiye Gory, bld. 40, Moscow, 119991 Russia; 4 Higher School of Economics, 20 Myasnitskaya st., Moscow, Russia

**Keywords:** Phylogeny reconstruction, Protein evolution, Algorithm, Open source software, Web interface

## Abstract

**Background:**

Many algorithms and programs are available for phylogenetic reconstruction of families of proteins. Methods used widely at present use either a number of distance-based principles or character-based principles of maximum parsimony or maximum likelihood.

**Results:**

We developed a novel program, named PQ, for reconstructing protein and nucleic acid phylogenies following a new character-based principle. Being tested on natural sequences PQ improves upon the results of maximum parsimony and maximum likelihood. Working with alignments of 10 and 15 sequences, it also outperforms the FastME program, which is based on one of the distance-based principles. Among all tested programs PQ is proved to be the least susceptible to long branch attraction. FastME outperforms PQ when processing alignments of 45 sequences, however. We confirm a recent result that on natural sequences FastME outperforms maximum parsimony and maximum likelihood. At the same time, both PQ and FastME are inferior to maximum parsimony and maximum likelihood on simulated sequences. PQ is open source and available to the public via an online interface.

**Conclusions:**

The software we developed offers an open-source alternative for phylogenetic reconstruction for relatively small sets of proteins and nucleic acids, with up to a few tens of sequences.

**Electronic supplementary material:**

The online version of this article (10.1186/s12859-018-2399-4) contains supplementary material, which is available to authorized users.

## Background

Phylogenetic reconstruction based on biological sequences is widely used in bioinformatics. Orthologous RNA and protein sequences are used to investigate the evolutionary relationships between taxonomic groups. Molecular biologists investigating protein families often reconstruct the phylogeny of these families to understand the evolutionary origins of important protein features, such as substrate specificity of enzymes.

Many software tools are available for phylogenetic reconstruction, and different tools often produce different results with the same input. At present, several types of phylogenetic algorithms are commonly used. The maximum parsimony (MP) criterion [1] informs the first type of algorithms; these algorithms rate trees using the number of mutations that are required to obtain a given set of sequences. The second class of algorithms are based on probabilistic models of sequence evolution and on the maximum likelihood (ML) criterion [2]. A specific variant of ML algorithms are quartet puzzle (QP) algorithms [3], where the criterion is not the likelihood itself, but the number of quartets of sequences such that the quartet topology induced by a given tree has the maximum likelihood among three possible topologies. The third class of algorithms uses evolutionary distance criteria. These distance-based algorithms vary widely, though the most popular are the neighbor-joining algorithm [4] and algorithms based on several varieties of the minimum evolution (ME) criterion.

This paper presents a new character-based algorithm based on a novel criterion PQ (for position-quartet) that resembles both MP and QP, but significantly differs from that. This new criterion is inspired by the fact that a correct tree often includes a number of branches that split sequences into groups with or without certain characters in certain alignment positions. It seems natural to count such branch-compatible positions and take their number as an optimality score for a tree.

However, the mentioned approach can hardly be applied as is, because branches close to the edges of the tree are more likely to produce a compatible position by chance, compared with branches more central to the tree. Thus, an optimization of this “position-branch” score would give an advantage to certain tree topologies, namely those having less “deep” branches. Moreover, in alignments of a substantial number of sequences, completely compatible positions are rather rare and counting a small number of such positions is not informative.

With these considerations in mind, our method counts branch-compatible positions, not in the whole tree, but instead in its four-leaf subtrees, which have only one branch each. The topology of a tree is known to be unambiguously determined by the topologies of its four-leaf subtrees. At the same time, many branch-compatible positions should occur in a four-sequence alignment. Hence, a correct tree should contain more alignment positions that support splits of the four-leaf subtrees, relative to an incorrect tree.

We propose the position-quartet (PQ) score, which counts the number of pairs of an alignment position and a quartet of sequences such that the position supports the subtree of the quartet. In the simplest variant (which is used for nucleic acid alignments) “support” means that one side of the quartet contains the same letter in the position and both letters on the other side are some other ones. The mentioned sides of any quartet are uniquely determined by the topology of the tree. If a position provides a “double” support (i.e., one letter in both sequences from one side and some other letter in both sequences from the other side of the quartet), then such position-quartet pair counts twice.

A refined version of the PQ score relies on the fact that in proteins, a specific feature of a clade may not be a single amino-acid residue at a certain position, but instead may represent a group of related residues at the position. This fact inspired us to use scoring matrices for amino acid residues. More precisely, a position supports a quartet, if the value of the scoring matrix on two letters on one side of the quartet is greater than on any two letters from different sides. The measure of support is the difference between the matrix value on the supported side and the maximum of matrix values across the split of the quartet. Again, if both sides of a quartet are supported by a position, the measure of support for such position-quartet pair is the sum of two measures. The overall score of a tree topology is the sum of these support measures over all positions of the alignment and all quartets of the tree.

In what follows, we report tests of our program with the BLOSUM62 matrix. We plan to compose a matrix designed especially for phylogenetic reconstruction with PQ, as BLOSUM62 was designed for protein alignment.

The PQ score resembles the parsimony score, as they are both summed over all positions of the alignment. They differ significantly, however, because the PQ score of a position is the sum of scores over all quartets of input sequences, while the parsimony score is the minimal number of mutations needed to produce the letters at a position *via* the given tree.

The criterion used in the quartet-puzzling (QP) method also resembles the PQ score. In the QP method, the main score is the number of quartets such that the tree-induced topology has the maximum likelihood among three possible quartet topologies. PQ and quartet-puzzling differ in two main respects: first, PQ uses the sum over all positions and all quartets instead of a simple count of quartets; second, PQ does not use the maximum likelihood criterion. In addition, the program TREE-PUZZLE [5], which is the only available realization of the quartet-puzzling method, yields a tree as a majority-rule consensus of many trees obtained by stepwise addition in randomized orders of input sequences, while PQ produces the tree with the highest found score.

Our tests show that PQ, MP, and QP yield different results. TNT [6] (a realization of MP) and PQ both produce fully resolved trees, and in all our tests, species trees are more distant from MP trees than they are from PQ trees, on average. TREE-PUZZLE (a realization of QP) usually produces unresolved trees, so it cannot be compared with PQ directly. Thus to compare PQ with QP we prepared a script that produces a resolved tree basing on draft trees generated by TREE-PUZZLE.

To evaluate the quality of phylogenetic reconstructions performed with PQ, we used natural instead of simulated protein sequences. With the available models of protein evolution, simulated sequence alignments differ from natural alignments in many respects. In the RAxML manual [7], A. Stamatakis writes, “…the current methods available for generation of simulated alignments are not very realistic. …Typically, search algorithms execute significantly less (factor 5–10) topological moves on simulated data until convergence as opposed to real data, i.e. the number of successful Nearest Neighbor Interchanges (NNIs) or subtree rearrangements is lower” and later: “…a program that yields good topological Robinson-Foulds distances on simulated data can in fact perform much worse on real data than a program that does not perform well on simulated data” (p. 60). Our results support the last statement. For example, ME outperforms ML on natural data but is inferior to ML on simulated data.

We used sequence alignments of orthologous proteins for testing; one protein per organism. We compared the reconstructed trees with species trees. We recognize that the actual tree of a given set of orthologous proteins may differ from the species tree because of horizontal gene transfer (HGT) and/or the loss of paralogs, but these deviations should not lead to incorrect conclusions when comparing phylogeny reconstruction methods. If a method reconstructs the actual tree better than another method, then the result from the first method will be closer to the species tree, in most cases. Exceptions to this trend are possible because reconstruction errors can by chance partly compensate for the difference between the real and species trees. Such exceptions, however, will produce just random noise, which is equally likely to improve the results from both methods. If such exceptions are rare, the resulting noise will not influence the comparison significantly. If not, and thus the noise is sufficiently large, then the comparison will yield statistically insignificant results.

On all the sets of alignments we tested, PQ shows a statistically significant (*p*<0.001) advantage over ML and MP. This indicates that the deviations between actual for each protein and species trees do not significantly affect our conclusions about the results of program comparison.

Testing phylogenetic programs on natural nucleotide sequences is a much more complicated task. We performed just two small tests on extractions from alignments of ribosomal RNA. These tests show that PQ performs well on nucleotide sequences, too.

We also performed tests on simulated protein and nucleic acid alignments. On the simulations, PQ is inferior to ML and MP. Also on the simulations ME and QP have less accuracy than MP and ML, in contrast to our tests on natural sequences. In our opinion, this primarily demonstrates a low quality of simulations made with current mutation models.

## Algorithm

### Tree score

Consider a multiple alignment of protein sequences and an unrooted binary phylogenetic tree with leaves labeled with the sequences of the alignment. We assume that more than three sequences are present. Let us denote the letter (*i.e.*, an amino acid residue or the gap symbol) in the *c*-th column of the *i*-th sequence of the alignment as *a*_*ic*_. Each four-element subset {*i*,*j*,*k*,*l*} of the sequences of the alignment can be divided into two two-element subsets following by the tree topology. We assume that this division is {*i*,*j*}∪{*k*,*l*}, which means that the tree contains at least one branch that separates *i* and *j* from *k* and *l*. We also fix an amino acid substitution matrix *S*(*a*,*b*), such as BLOSUM62.

The tree score *Q* is calculated using the following formula:

$$Q = \sum\limits_{c} \sum\limits_{q} Q_{cq} $$

where *c* accounts for all columns of the alignment, *q* accounts for all quartets {*i*,*j*,*k*,*l*} of sequences such that *a*_*ic*_, *a*_*jc*_, *a*_*kc*_, *a*_*lc*_ are residues (not gaps), and *Q*_*cq*_ (called the position-quartet score or the PQ score) is given by the following formula:

1 $$ Q_{cq} = \max\left(S(a_{ic},a_{jc}) - X_{cq}, 0\right) + \max\left(S(a_{kc},a_{lc}) - X_{cq},0\right)  $$

where

$$X_{cq} = \max \left(S\left(a_{ic},a_{kc}\right), S\left(a_{ic}, a_{lc}\right), S\left(a_{jc},a_{kc}\right), S\left(a_{jc}, a_{lc}\right)\right) $$

For example, if the matrix *S*(*a*,*b*) is diagonal, with all diagonal elements equal to 1 and other elements equal to 0 (which is a natural choice for nucleic acid sequences), then the PQ score *Q*_*cq*_ is equal to:

- 0 if all four letters *a*_*ic*_,*a*_*jc*_,*a*_*kc*_,*a*_*lc*_ are different;
- 0 if the intersection of two sides of the split quartet, {*a*_*ic*_,*a*_*jc*_} and {*a*_*kc*_,*a*_*lc*_}, is not empty (particularly if all four letters are the same);
- 1 if *a*_*ic*_=*a*_*jc*_ while *a*_*ic*_≠*a*_*kc*_, *a*_*kc*_≠*a*_*lc*_, and *a*_*ic*_≠*a*_*lc*_;
- 1 if *a*_*kc*_=*a*_*lc*_ while *a*_*ic*_≠*a*_*kc*_, *a*_*ic*_≠*a*_*jc*_, and *a*_*jc*_≠*a*_*kc*_;
- 2 if *a*_*ic*_=*a*_*jc*_ and *a*_*kc*_=*a*_*lc*_, but *a*_*ic*_≠*a*_*kc*_.

We also implemented a generalized variant of the PQ score. It is based on the idea that a quartet that has two pairs of similar letters of both its sides should “cost” more than just a sum of contributions of two sides. Thus it seems natural to multiple the score *Q*_*cq*_ of a position-quartet pair (*c*,*q*) by a certain number if both sides of the quartet contribute positively to the score.

More precisely, let *α* be any positive number. Replace the above formula (1) for *Q*_*cq*_ with the following:

2 $$ Q_{cq} = \left\{ \begin{array}{l} 0 \text{, if}\ S(a_{ic},a_{jc})\le X_{cq} \text{ and}\ S(a_{kc},a_{lc})\le X_{cq}\\ S(a_{ic},a_{jc}) - X_{cq} \text{, if}\ S(a_{ic},a_{jc})>X_{cq} \text{ and}\ S(a_{kc},a_{lc})\le X_{cq}\\ S(a_{kc},a_{lc}) - X_{cq} \text{, if}\ S(a_{ic},a_{jc})\le X_{cq} \text{ and}\ S(a_{kc},a_{lc})>X_{cq}\\ \alpha \left(S(a_{ic},a_{jc}) + S(a_{kc},a_{lc}) - 2X_{cq}\right), \\ \text{\qquad if}\ S(a_{ic},a_{jc})>X_{cq} \text{ and}\ S(a_{kc},a_{lc})>X_{cq} \end{array}\right.  $$

This formula reduces to (1) if *α*=1.

Our implementation of PQ includes two ways of accounting gaps, in addition to the default variant in which gaps are ignored. The gap symbol is treated as an additional letter in both variants. One variant makes no difference between gaps and other letters, which denote amino acid residues or nucleotides, and the other accounts for *Q*_*cq*_ only if the quartet *q* in the position *c* includes one gap at most.

#### Normalized tree score

Together with the tree score described above, the normalized tree score is computed as follows. For each quartet of input sequences *q* and each position *c* the maximum position-quartet score $Q^{m}_{cq}$ is calculated as the maximum value of the above-described *Q*_*cq*_ scores among all three possible splits, regardless of the split realized in the tree. We define *Q*^*m*^ as the sum of all $Q^{m}_{cq}$. Note that *Q*^*m*^ does not depend on tree topology, but depends only on the input alignment. Finally, we define the normalized tree score *S* as the ratio *Q*/*Q*^*m*^. If the input alignment is fixed, then *S* is proportional to *Q*; *S* simultaneously gives a more-objective indicator for the tree-reconstruction quality when considering various alignments. Indeed, *Q* depends on the total numbers of quartets and positions, while *S* is the fraction of position-quartet pairs that support the tree and thus does not directly depend on the size of an input alignment. Tests show that both values negatively correlate with distance from the inferred tree to the reference tree, but for all tested sets the correlation coefficient between *S* and the distance is higher in absolute value.

### Search algorithms

For a given alignment, the tree with the highest score must be identified. An exact solution requires factorial time, so we used several standard heuristics to select a tree scored nearly the highest. It is possible that trees with several topologies have the same highest score, in this case, the program returns the one found first.

#### Stepwise addition

This heuristic fixes the order of the input sequences. For the first four sequences, it finds the tree with the best score, which only requires checking three trees. Then the fifth sequence is added, and the best tree is chosen from the trees with five leaves such that their subtrees with the first four leaves coincide with the tree found at the first step. Sequences are added in this manner until a tree corresponding to the entire set of sequences is obtained.

#### Multiple stepwise addition

The process of stepwise addition is repeated several times while changing the input order of sequences with random shuffling. The result is the best-scoring tree among all obtained trees.

#### NNI hill climbing

From an initial tree, such as the result of stepwise addition, this heuristic performs all possible nearest-neighbor interchanges (NNI) [8], one by one. If the current NNI yields a tree with a higher score, then that tree is processed again. This heuristic repeats until all NNIs of the current tree yield trees with scores not greater than the score of the current tree.

#### NNI Monte Carlo optimization

An initial temperature *T*=*T*_ini_ is set, *T*_ini_=1000 by default, and *K*=12000000. Only the ratio *K*/*T* is significant, so we set *K* to be large enough to allow *T* to be expressed as an integer. Then all possible NNIs are performed one by one in an initial tree. If the current NNI gives a tree with a score *Q*_new_ that is greater than the score *Q*_old_ of the current tree, then the procedure is repeated with the new tree. If *Q*_new_<*Q*_old_, then the new tree is next processed with the probability:

$$P = \exp \left({K\over T}\cdot{Q_{\text{new}} - Q_{\text{old}}\over Q_{\text{old}}}\right) $$

and with the probability 1−*P* the next NNI is performed on the old tree. *T* is reduced by *T*_ini_/*N* after each step, where *N* is a parameter, *N*=1000 by default. The process stops when *T* reaches zero. The tree with the highest score among all tested is output.

#### SPR hill climbing

SPR hill climbing is analogous to NNI hill climbing, but uses subtree pruning and regrafting (SPR [9]) instead of NNI.

## Materials and methods

### Compared software

We compared results of our program with implementations of four well-known algorithms for phylogenetic reconstruction. These algorithms are: maximum parsimony (MP) implemented in TNT 1.1 [6], maximum likelihood (ML) implemented in RAxML 8.2.8 [7, 10], balanced mimimum evolution (ME) implemented in FastME 2.1.5 [11] and quartet puzzle (QP) implemented in TREE-PUZZLE 5.2 [5].

For MP the parameters are as follows:

- Program: TNT
- Result: RAxML_parsimonyTree
- Search strategy: “mult”, which means several rounds of randomized stepwise addition of sequences followed by search using tree bisection and reconnection (TBR).

For our ML tests, we used the PROTGAMMAAUTO model of RAxML for amino acid sequences and GTRGAMMA model for nucleotide sequences. All other parameters remained set at default values. We took the so-called “bestTree” from the output of RAxML, as the result for comparison. The parameters for ML are as follows:

- Program: RAxML 8.2.8
- Result: RAxML_bestTree
- Amino acid substitution model: PROTGAMMAAUTO. This involves automatic model choice and using the gamma distribution of rates; see [7] for details.
- Nucleotide substitution model: GTRGAMMA.
- Search strategy: starting with MP tree several SPR steps are performed with the radius (*i.e.* the number of nodes away from the original pruning position) determined automatically by RAxML.

For ME tests, we used FastME 2.1.5 with the default parameters:

- Program: FastME v2.1.5.
- Amino acid substitution model for distance calculation: LG, gamma rate variation parameter (alpha) equals 1, do not remove sites with gaps.
- Initial tree: BIONJ (see [11] for details).
- Search strategy: NNI and SPR postprocessing.

For QP tests, we used the program TREE-PUZZLE 5.2. This program produces an unresolved tree in general case, which makes impossible a direct comparison with other programs producing resolved (binary) trees. Thus we implemented a script that takes so-called “puzzling step trees” generated by TREE-PUZZLE and inputs it to the program *consense* of PHYLIP [12] package. The latter is able to produce a resolved consensus of a number of trees with so-called extended majority rule. The number of puzzling steps was set to 100, other parameters were by default:

- Program: a pipeline from TREE-PUZZLE 5.2 to *consense*.
- Substitution model: auto; parameter estimates: approximate.
- Rate of site heterogeneity: uniform.
- Approximate quartet likelihood.
- Number of puzzling steps: 100.
- List puzzling step trees.
- Consensus type: majority rule (extended)

### Data sets of protein alignments

We used three sets of organisms: 25 Metazoa species, 45 Fungi species and 45 Proteobacteria species.

The fungal and proteobacterial species were selected trying to maximize the total number of common Pfam [13] families in their proteomes. Pfam families consist of evolutionary domains, which are segments of proteins whose evolution included only point mutations and small insertions or deletions, without large rearrangements. The evolution of these domains can be studied by analyzing their alignments.

The metazoan species were chosen with the NCBI taxonomy in mind: the goal was a set of popular organisms, with many sequenced proteins and a fully resolved taxonomic tree.

For each set we found as many orthologous groups of protein domains as was possible, using the procedure described in [14]. In brief, this procedure uses the following instructions.

From a set of species, take all Pfam families that are present in all species. For each family, take all sequences of protein domains of this family from all species. Then construct pairwise global alignments of the sequences from different species and compute the alignment scores. Finally, find the best bidirectional hits, which are pairs of domains from different species in which each member of the pair has the maximum alignment score with the other member when compared with all other domains of the same species. An orthologous group is defined as a set of domains, one from each species, such that each pair of the domains forms a best bidirectional hit.

The organisms are listed in Additional file 1, and the sequences of orthologous groups are in Additional file 2.

To examine the relative effectiveness of the programs when analyzing differently sized alignments, we used alignments of subsets of sequences from each orthologous group in addition to alignments of entire orthologous groups. We thus tested the programs on nine alignment datasets, as listed in Table 1.

**Table 1:** Alignment datasets

Name	Number of alignments
Metazoa-10	1499
Metazoa-15	1283
Fungi-15	1191
Proteobacteria-15	784
Metazoa-25	970
Fungi-30	1004
Proteobacteria-30	783
Fungi-45	827
Proteobacteria-45	780

The name of each set consists of the taxon name and the number of sequences in each alignment of the set

Each metazoan orthologous group was randomly split into 10 and 15 sequences; each fungal or proteobacterial orthologous group was split into 15 and 30 sequences. All the sets of sequences so obtained were aligned using Muscle 3.8.31 [15].

An alignment was removed from the dataset if: (i) it contains two or more identical sequences, or (ii) the distance matrix (generated by the *protdist* program of the PHYLIP package) contains negative distances, meaning that some sequences are too distant so that the distance likelihood function has no maximum. This explains why, for example, the Metazoa-25 dataset contains fewer alignments than the Metazoa-15 dataset.

### Comparison procedure for protein alignments

To compare two fully-resolved (binary) trees for the same set of species, we use the normalized Robinson–Foulds distance [16], which is the number of different splits in the two trees, divided by the total number of splits in the trees. This value remains between 0 and 1.

A species tree was created for each dataset. For the 25 metazoa, we designed this tree to be unique, as supported by the NCBI Taxonomy, since the 25 species were selected to ensure this. For the 45 fungi, the species tree is the consensus of all trees that were built by all four of the MP, ML, ME and QP methods using all alignments of 45 fungal domains. This consensus was created with the program *consense* of PHYLIP package using the “Majority rule extended” option, which yields a binary consensus tree. The same procedure was used for the 45 proteobacteria.

For the other datasets we studied, namely Metazoa-10, Metazoa-15, Fungi-15, Fungi-30, Proteobacteria-15 and Proteobacteria-30, species trees were obtained by restricting the corresponding complete tree to the appropriate subset of organisms.

All three of the complete species trees are included in Additional file 2 in Newick format and as PNG images.

For each alignment, we computed the normalized Robinson–Foulds distances between the corresponding species tree and the trees created by the five methods: PQ, MP, ML, ME and QP.

For each alignment dataset, we compared the results from PQ with results from MP, ML, ME, and QP. To compare PQ with, for instance, MP, we counted the number of alignments for which the distance from the PQ tree to the species tree is less than the distance from the corresponding MP tree to the species tree. We also counted the number of alignments for which the distance from the PQ tree is greater than the distance from the MP tree. These two numbers were then compared by the sign test. If the *p*-value is less than 0.001, then one of the compared methods is judged to be more effective for the present dataset.

As a reference for fungal and proteobacterial alignments, we may use the consensus of trees created by any one program alone with almost the same results. All three consensus trees are close to each other. For Fungi, the maximum normalized Robinson–Foulds distance 2/42≈0.048 occurs between the MP and ML consensus trees, meaning that each tree contains two splits of 42 that are not presented in another tree. For Proteobacteria, the maximum distance 8/42≈0.19 occurs between the ME and QP consensus trees. The comparison results depend only slightly on the choice of the reference tree. For example, comparing PQ with ML on Proteobacteria-30, the result is 430/186 using the overall consensus as a reference, *i.e.*, in 430 cases the PQ reconstruction is closer to the reference and in 186 cases it is farther. Compare these values with 428/195 using the ML consensus, 429/181 using the ME consensus, 421/183 using the MP consensus, and 431/194 using the QP consensus; these are all quite close to each other.

### Datasets of nucleic acid alignments and comparison procedure for them

To produce a good reference dataset of nucleic acid alignments is a much more complicated task comparing to the same one for protein alignments. We decided to perform a rather small test to check the ability of PQ to reconstruct phylogeny from a set of nucleic acid sequences.

For 45 fungi and 45 proteobacteria that are involved in the protein test, we downloaded their small ribosomal RNA from the database Silva [17]. We aligned these two sets of RNA sequences by Muscle, then excluded redundant sequences (there are two pairs of completely identical rRNA sequences in the fungal set), also, we removed all sites represented by only one sequence. The resulting alignments consist of 43 sequences and 1853 columns for Fungi and of 45 sequences and 1666 columns for Proteobacteria, these alignments are available in Additional file 3. Then 100 times for Fungi and 100 times for Proteobacteria we performed the following procedure: random selection of a number *N* from the range 300 to 800; random selection of 15 species and *N* columns from the alignment; composing an artificial alignment from these rows and columns. The resulting set of 200 artificial subalignments was used for testing programs. These subalignments and trees inferred from them are available in Additional file 3. We used restrictions of our species trees to corresponding species subsets as reference trees.

### Simulated alignments

Amino acid simulated alignments were extracted from raw data to the paper [18] from Dryad Digital Repository [19]. From there we used 500 “reference” alignments from the folder “simulation/30taxa” in the archive rawData.zip. According to that paper, “30-sequence multiple sequence alignments were simulated using Artificial Life Framework (ALF) [20]. The sequence length was drawn from a Gamma distribution with parameters *k*=2.78, *θ*=133.81. Sequences were evolved along 30-taxa birth–death trees (with parameters *λ*=10*μ*) scaled such that the distance from root to deepest branch was 100 point accepted mutation (PAM) units. Characters were substituted according to WAG substitution matrices [21], and insertions and deletions were applied at a rate of 0.0001 event/PAM/site, with length following a Zipfian distribution with exponent 1.821 truncated to at most 50 characters (default ALF parameters).”

Five hundred nucleotide 15-sequence alignments were simulated using *phylosim* R package [22]. The trees for simulations were created by *rtree* function from the phylosim package with parameters by default, which means branch lengths uniformly distributed in interval 0 to 100 PAM. The length of the initial sequence was chosen uniformly from 300 to 800. Characters were substituted according to GTR substitution model with a mutation rate heterogenity modeled according to a Gamma distribution with the shape parameter of 4.5 and the fraction of invariant sites of 0.5. Insertions and deletions were applied at a rate of 0.0045 event/PAM/site, with the maximum length of 4. The simulated nucleotide alignments, the trees used for simulations, and the trees inferred from the alignments are available in Additional file 4.

## Implementation

We implemented PQ in a command-line application written in ANSI C. The source code, an executable file for Windows, and a brief user manual are available at http://mouse.belozersky.msu.ru/software/pq/.

The program takes an alignment in Fasta format as input and outputs an unrooted tree with no branch lengths in Newick format. Users may select a number of parameters, among them the file with the scoring matrix, the positive integer value of *α*, and the optimization strategy to be used. Further details are available in the online user manual.

A web interface is available at http://mouse.belozersky.msu.ru/tools/pq/. It allows the reconstruction of phylogeny from alignments of up to 100 sequences using any optimization strategy except for SPR. For user convenience, the web interface returns an unrooted tree without branch lengths along with a rooted phylogram that has the same topology. Branch lengths are computed by the program *proml* in the PHYLIP package. The resulting tree with branch lengths is rooted to its midpoint. The program *drawgram* in PHYLIP is used to generate an image of the tree.

## Results and discussion

### Time and memory complexity

The time complexity of PQ with parameters by default, i.e., using 10-fold stepwise addition followed by gradient NNI search, is *C*_1_*N*^4^*L*+*C*_2_*N*^5^, where *N* is the number of sequences in the input alignment, *L* is the number of informative (not completely conserved) sites in the alignment, and *C*_1_ and *C*_2_ are coefficients that do not depend on *N* or *L*. During the stepwise addition, calculation of *Q*_*cq*_ for all alignment columns *c* and all quartets *q* requires *O*(*N*^4^*L*) operations. After that ${N\choose 4}$ sums over columns can be stored in memory. Stepwise addition implies *N*−4 steps of *O*(*N*^4^) operations each, because each step requires testing, in average, (2*N*−3)/2 branches and testing each branch requires calculations with *O*(*N*^3^) quartets (not *O*(*N*^4^) because the fourth member of each quartet is fixed, it is the added leaf). During the NNI search, each round implies testing *N*−3 branches, with calculations with *O*(*N*^4^) stored quartets for each branch.

The memory complexity of the program is proportional to ${N\choose 4}$.

Testing on fungal alignments shows that the performance of PQ with default parameters takes for a 30-sequence alignment in average 26 times more time and for a 45-sequence alignment 223 times more time comparing with a 15-sequence alignment. This approximately coincides with the *N*^5^ rule.

SPR requires more computation time than NNI and the difference grows dramatically with the number of sequences. For alignments of the Metazoa-10 dataset, SPR takes on average of 1.3 times more time than NNI hill climbing and 2.5 times more time than single stepwise addition; for Proteobacteria-45, the values are 30 times and 210 times, respectively. Theoretical considerations give the sixth power dependence of time with respect to the number of sequences for one round of an SPR search. However, the average number of the rounds also may grow with the sequence number and the rule of this growth is hard to predict theoretically.

Comparing with other programs, the fastest one is FastME. The work of FastME with one 45-sequence alignment takes (at our computer) in average 0.13 s. For TNT this time is 0.23 s, for PQ (with parameters by default) is 12 s, for TREE-PUZZLE is 100 s and for RAxML is 430 s. Among these programs, PQ has the worst time dependence on the number of sequences. A rough extrapolation shows that PQ would work faster than RAxML up to approximately 150 sequences in the input data.

### Tree scores and distances to the species tree

Table 2 lists the mean normalized tree scores *S*, mean normalized Robinson–Foulds distances to the species trees *D*, and correlation coefficients: *r*_*SD*_ between the scores and the distances, *r*_*SL*_ between the scores and the lengths of alignments, and *r*_*DL*_ between the distances and the lengths. All data are for trees obtained through NNI hill climbing using the BLOSUM62 scoring matrix. The parameter *α* was equal to 1, and gaps were ignored. We also tested other values of *α*, namely 2, 3, 5 and 10, and we took gaps into account, but neither of those improved accuracy, so we omit those results from this paper.

**Table 2:** Mean relative tree scores (<*S*>), mean normalized Robinson – Foulds distances to the species trees (<*D*>) and the correlations coefficients: between scores and distances (*r*_*SD*_), between scores and alignment lengths (*r*_*SL*_), and between distances and lengths (*r*_*DL*_)

Dataset	<*S*>	<*D*>	*r*_*SD*_	*r*_*SL*_	*r*_*DL*_
Metazoa-10	0.9919	0.345	− 0.40	0.29	− 0.21
Metazoa-15	0.9901	0.388	− 0.44	0.37	− 0.25
Fungi-15	0.9915	0.329	− 0.42	0.38	− 0.31
Proteobacteria-15	0.9816	0.564	− 0.27	0.39	− 0.03
Metazoa-25	0.9900	0.418	− 0.39	0.42	− 0.25
Fungi-30	0.9908	0.415	− 0.43	0.45	− 0.33
Proteobacteria-30	0.9779	0.682	− 0.25	0.42	− 0.15
Fungi-45	0.9912	0.445	− 0.48	0.47	− 0.33
Proteobacteria-45	0.9762	0.739	− 0.29	0.43	− 0.18

Optimization strategy was 10 times repeated stepwise addition followed by NNI hill climbing, the scoring matrix was BLOSUM62

Turning to an analysis of the distances between the reconstructed and species trees, first, notice the difference between fungi and proteobacteria datasets. Trees reconstructed from proteobacterial alignments are on average much more distant from the corresponding species tree than are trees reconstructed from fungal alignments. This divergence may be explained by HGT, which is rather frequent among bacteria. Due to HGT, the real phylogeny of a protein family may differ slightly from the phylogeny of the corresponding organisms, and this difference will increase the distances we consider. Other causes likely contribute to this divergence as well; the lower values of *S* for proteobacterial datasets hint that specific features of proteobacterial alignments make phylogeny reconstruction more difficult. The correlation *r*_*SD*_ between the normalized scores and distances to the species trees is rather stable for all fungal and metazoan datasets and is practically independent of the size of the alignments. For proteobacterial datasets, the values of *r*_*SD*_ are also stable with respect to alignment size, but they are significantly lower than those for eukaryotic datasets.

### Optimization strategies

For all alignments, we reconstructed phylogenies with PQ using the following six optimization heuristics: single stepwise addition, stepwise addition with randomized order repeated tenfold, 100-fold repeated stepwise addition, NNI hill climbing, NNI Monte Carlo search, and SPR hill climbing. Each NNI and SPR search started with the best-scoring result of the tenfold repeated stepwise addition. We measured the frequency at which each heuristic reaches the maximum tree score of the six trees, and how frequently the heuristic produces the minimum Robinson–Foulds distance to the species tree. The results are listed in Tables 3 and 4.

**Table 3:** Percents of alignments for which different search strategies reach a maximum tree score

Dataset	1SA	10SA	100SA	NNI HC	NNI MC	SPR
Metazoa-10	61.4%	99.3%	99.9%	99.5%	100%	99.8%
Metazoa-15	42.3%	92.2%	99.8%	97.7%	99.4%	99.1%
Fungi-15	41.6%	91.9%	99.7%	98.7%	99.7%	99.4%
Proteobacteria-15	25.5%	73.5%	97.2%	93.5%	98.2%	97.1%
Metazoa-25	22.6%	72.0%	96.6%	92.4%	95.1%	98.9%
Fungi-30	8.7%	42.3%	87.1%	85.8%	92.0%	97.8%
Proteobacteria-30	1.4%	11.4%	41.0%	57.3%	70.9%	93.4%
Fungi-45	1.6%	13.3%	48.0%	62.8%	75.1%	96.1%
Proteobacteria-45	0.0%	0.4%	4.4%	27.4%	37.2%	88.5%

1SA, 10SA and 100SA are for single, 10 times and 100 times repeated stepwise addition, respectively; NNI HC is for NNI hill climbing, NNI MC is for NNI Monte Carlo search

**Table 4:** Percents of alignments for which different search strategies reach minimum Robinson – Foulds distance to the species tree

Dataset	1SA	10SA	100SA	NNI HC	NNI MC	SPR
Metazoa-10	85.4%	91.4%	91.5%	91.3%	91.5%	91.7%
Metazoa-15	80.3%	84.8%	85.1%	85.3%	85.0%	85.3%
Fungi-15	75.1%	83.4%	83.7%	83.8%	83.5%	83.7%
Proteobacteria-15	71.0%	80.9%	81.5%	80.1%	81.0%	81.1%
Metazoa-25	70.8%	77.4%	78.5%	78.5%	78.8%	78.2%
Fungi-30	50.2%	63.3%	65.8%	65.8%	65.3%	65.5%
Proteobacteria-30	42.5%	55.6%	57.7%	53.8%	57.2%	58.5%
Fungi-45	37.7%	49.1%	49.6%	49.8%	49.7%	52.5%
Proteobacteria-45	31.8%	38.3%	39.9%	43.8%	40.9%	46.0%

Notation is the same as in Table 3

We expected and found that more-complicated optimization algorithms are required to obtain a maximum possible tree score for alignments of more sequences. Less expected, we found that the difference between complicated and simple optimization algorithms is less for distance to the species tree than it is for tree scores. This likely indicates that the tree score well distinguishes a tree that is far enough from the real tree from a tree that is close to the real tree, but that the score often fails to choose among two nearly correct trees. This trend resembles results obtained by Takahashi and Nei [23] in tests with MP, ML, and ME scores using simulated data.

Analysis of the results presented in Table 3 suggests that proteobacterial alignments have some features that make phylogenetic reconstruction harder than it is with eukaryotic alignments. Note that the data in Table 3 is independent of the species tree and, therefore, does not depend directly on possible HGTs. Nevertheless, with prokaryotic alignments each search strategy reaches the highest tree score less frequently than with eukaryotic alignments of the same number of sequences. This result is in accordance with the lower normalized tree scores for proteobacterial alignments. HTG from taxons other than Proteobacteria may make tree topology more complicated, and this is one possible explanation of the phenomenon.

Another feature that complicates the reconstruction lies in the shorter average length of proteobacterial protein domains, as compared with eukaryotic protein domains. For example, the median alignment length in Fungi-45 is 264, and in Proteobacteria-45 is 160. The normalized tree score correlates well with the length of the alignment as it is shown in Table 2. But the domain length is not the only factor complicating reconstructions of proteobacterial phylogeny. To check this, we extracted alignments of medium length, namely all alignments of the length between 161 and 263, from Fungi-45 and Proteobacteria-45. These datasets include nearly equal numbers of such alignments: 222 from Fungi-45 and 217 from Proteobacteria-45. For these medium-length alignments, the difference between Fungi and Proteobacteria is also impressive. For example, 100-fold stepwise addition gives a maximum score among scores that can be reached with at least one of the heuristics for only 8, which is 3.7*%*, proteobacterial medium-length alignments and for 96, which is 43.2*%*, fungal medium-length alignments. It means that even working with alignments of the approximately same length, the simple search strategy produces the same result as more complicated strategies much less frequently in case of proteobacteria comparing with the case of fungi.

The behavior of the mean normalized score confirms this length-independent relative complexity of proteobacterial alignments. For fungal medium-length alignments mean value of *S* is 0.9899, which is lower than that for the total set of fungal 45-sequence alignments (0.9912) but higher than that for proteobacteial medium-length alignments, 0.9795.

### Comparison with other programs on protein alignments

We examined the results of NNI hill climbing to compare PQ with other software, and list the results in Tables 5, 6, 7, and 8.

**Table 5:** Average Robinson – Foulds distances between the species trees and reconstructions by the programs

Dataset	PQ	ME	ML	MP	QP
Metazoa-10	0.345	0.379	0.390	0.433	0.357
Metazoa-15	0.388	0.417	0.424	0.475	0.401
Fungi-15	0.329	0.355	0.391	0.417	0.335
Proteobacteria-15	0.564	0.584	0.620	0.633	0.574
Metazoa-25	0.418	0.441	0.440	0.515	0.437
Fungi-30	0.415	0.421	0.444	0.486	0.417
Proteobacteria-30	0.682	0.697	0.718	0.747	0.693
Fungi-45	0.445	0.438	0.457	0.512	0.452
Proteobacteria-45	0.739	0.744	0.761	0.790	0.744

**Table 6:** Numbers of “good” reconstructions

Dataset	Threshold	PQ	ME	ML	MP	QP
Metazoa-10	0.143	192	145	152	111	166
Metazoa-15	0.25	297	267	253	129	262
Fungi-15	0.167	166	143	108	71	161
Proteobacteria-15	0.417	143	126	81	71	127
Metazoa-25	0.273	188	169	173	61	147
Fungi-30	0.296	206	208	182	96	185
Proteobacteria-30	0.593	186	166	127	78	163
Fungi-45	0.357	198	236	211	108	187
Proteobacteria-45	0.643	152	134	110	57	128

The column “Threshold” contains first quartils of Robinson – Foulds distances between PQ trees and species trees, for each set. Numbers in other columns are numbers of trees reconstructed by each method whose distance to the corresponding species tree is less than the threshold. Numbers in PQ column are less than 1/4 of total volumes of the sets because the distance can take only few possible values

**Table 7:** Numbers of “bad” reconstructions

Dataset	Threshold	PQ	ME	ML	MP	QP
Metazoa-10	0.571	193	239	248	317	210
Metazoa-15	0.5	320	375	402	487	333
Fungi-15	0.417	278	336	413	486	287
Proteobacteria-15	0.667	184	213	250	297	189
Metazoa-25	0.545	203	247	248	371	213
Fungi-30	0.518	223	235	297	355	212
Proteobacteria-30	0.778	173	210	252	290	197
Fungi-45	0.524	205	202	255	344	200
Proteobacteria-45	0.833	169	172	202	262	172

The column “Threshold” contains third (higher) quartils of Robinson – Foulds distances between PQ trees and species trees, for each set. Numbers in other columns are numbers of trees reconstructed by each method whose distance to the corresponding species tree is greater than the threshold. Numbers in PQ column are less than 1/4 of total volumes of the sets because the distance can take only few possible values

**Table 8:** Pairwise comparison of PQ with ME, ML, MP, and QP

Dataset	ME	ML	MP	QP
Metazoa-10	**466/240**	**530/261**	**683/189**	**342/255**
Metazoa-15	**483/291**	**566/300**	**758/184**	**370/270**
Fungi-15	**467/275**	**638/209**	**730/181**	352/302
Proteobacteria-15	**283/188**	**403/143**	**417/127**	236/184
Metazoa-25	**432/266**	**458/270**	**676/113**	**413/220**
Fungi-30	412/390	**525/313**	**687/186**	396/360
Proteobacteria-30	**353/233**	**430/186**	**530/119**	**338/232**
Fungi-45	**303/406**	**412/315**	**589/152**	382/306
Proteobacteria-45	350/273	**426/217**	**550/128**	347/279

The number before “/” in each cell is the number of alignments for which PQ result is closer to the species tree, the second number is the number of alignments for which PQ result is more distant from the species tree. Statistically significant (*p*<0.001) results are in boldface

Table 5 contains the average distances to species trees, for each dataset and each tested method.

Table 6 contains the numbers of alignments producing relatively good results. As thresholds for this “relative goodness” we chose the lower quartiles of RF distances among trees built by PQ for each particular dataset, thus these numbers for PQ are always close to 25% of the dataset volume. The percents are not equal to 25% exactly because RF distance takes a limited number of possible values. For example, for Metazoa-10 the lower quartile of RF distances between PQ trees and reference trees is 1/7, i.e. the lowest possible nonzero value. Thus for this data set, the percent of good results is equal to the percent of perfect results, i.e. alignments for which the inferred phylogeny coincides with the real phylogeny. For 15-species data sets, the percents of perfect results are much lower, 1.2 to 2.3*%* for Metazoa-15, 1.3 to 4.1*%* for Fungi-15 and 0 to 0.3*%* for Proteobacteria-15. For other datasets, there are almost no perfect results of any program.

Table 7 contains the percents of alignments producing relatively bad results. Thresholds are the higher quartiles of RF distances among trees built by PQ for each dataset.

Table 8 contains the results of pairwise comparisons of PQ with ME, ML, MP, and QP, as detailed in Materials and Methods. We conclude from Table 8 that PQ reconstructs phylogeny more accurately than do ML and MP for all the datasets we tested. However, there is a significant point to note about relative accuracy of PQ and ML. The distances between ML trees and species trees correlate with lengths of alignments stronger, comparing with distances between PQ trees and species trees. For example, for Fungi-30 the correlation coefficient is − 0.46 for ML trees and − 0.33 for PQ trees, for Proteobacteria-30 − 0.22 and − 0.15, respectively. Regarding only alignments of Fungi-45 with the length greater than 550, ML has a statistically significant advantage over PQ. Namely among 64 such alignments, for 47 the ML tree is closer to the species tree and only for 11 is more distant than the PQ tree. For all other sets the difference between ML and PQ for long (length > 550) alignments is not significant, but the ratio of two numbers, “ML better” to “PQ better” is always less for long alignments than for short ones. It is not completely clear if this effect is due to the alignment length itself or is related to some features of large proteins.

For sets with alignments of 10, 15, and 25 sequences, PQ is more accurate than ME. The same is correct for the Proteobacteria-30 set. For two sets, Fungi-30 and Proteobacteria-45, the difference between PQ and ME is not statistically significant, and for Fungi-45 ME outperforms PQ.

Note that the advantage of ME over both ML and MP accords with G. Gonnet’s results from only, as far as we know, testing phylogeny reconstruction methods on large natural datasets [24]. The commonly held opinion that ML is more accurate than distance-based methods is probably based on tests with simulated alignments, which may differ significantly from alignments of natural sequences.

PQ is more accurate than QP for all metazoan sets, and also for Proteobacteria-30. For other sets, the difference between PQ and QP is not statistically significant, but PQ is always slightly better.

### Comparison with other programs on nucleotide alignments

Tables 9 and 10 demonstrate results of the five programs on subalignments of rRNA sequences. All programs show medium results for subalignments of fungal 18S rRNA and poor results (average distance to reference is about 0.5) for proteobacterial subalignments. For both sets PQ shows slightly better results comparing with ME and QP and significantly better results comparing with ML and MP. For fungal subalignments ML shows a greater dependence on the subalignment length than other programs, which is in accordance with the same phenomenon for protein alignments.

**Table 9:** Results of the programs on 100 extractions from the alignment of fungal 18S rRNA

Value	PQ	ME	ML	MP	QP
<*D*>	0.20	0.21	0.23	0.28	0.22
*r*_*DL*_	− 0.17	− 0.21	− 0.37	− 0.25	− 0.21
Perfect	9	9	4	4	6
Bad	18	23	29	45	21
PQ is better	NA	33	46	64	28
PQ is worse	NA	22	26	14	16
*P*-value	NA	0.17	0.024	8·10^−9^	0.1

The row <*D*> contains average Robinson – Foulds distances to the species tree, the row *r*_*DL*_ contains the correlation coefficient between distance and alignment length. “Perfect” are numbers of inferred trees that coincide with the species tree. “Bad” are numbers of inferred trees whose distance from the species tree is greater than 0.25. “PQ is better” and “PQ is worse” are numbers of trees whose distance from the species tree is, respectively, greater or less than the same distance of the tree inferred by PQ, “*P*-value” is the *p*-value of comparison the least two numbers by the sign test

**Table 10:** Results of the programs on 100 extractions from the alignment of proteobacterial 16S rRNA

Value	PQ	ME	ML	MP	QP
<*D*>	0.38	0.43	0.51	0.50	0.40
*r*_*DL*_	− 0.26	− 0.28	− 0.16	− 0.09	− 0.21
Good	27	21	9	12	24
Bad	14	25	45	41	18
PQ is better	NA	51	76	74	34
PQ is worse	NA	7	8	9	14
*P*-value	NA	2·10^−9^	5·10^−15^	8·10^−14^	0.01

“Good” are numbers of inferred trees whose distance from the species tree is less than 0.25. “Bad” are numbers of inferred trees whose distance from the species tree is greater than 0.5. Other notations are the same as in Table 9

### Comparison with other programs on simulated alignments

Tables 11 and 12 demonstrate results of the five programs on simulated alignments. On amino acid simulations, the best results are demonstrated by ML, MP is much worse, PQ and QP are approximately equal and slightly worse than MP and the worst is ME.

**Table 11:** Results of the programs on 500 simulated amino acid alignments

Value	PQ	ME	ML	MP	QP
<*D*>	0.144	0.165	0.111	0.133	0.136
*r*_*DL*_	− 0.34	− 0.49	− 0.50	− 0.48	− 0.52
Perfect	15	13	65	24	19
Good	156	115	316	181	172
Bad	106	146	30	96	96
PQ is better	NA	248	53	167	188
PQ is worse	NA	145	347	227	198
*P*-value	NA	2·10^−7^	5·10^−54^	0.003	0.65

The row <*D*> contains average Robinson – Foulds distances between inferred trees and reference trees. “Good” are numbers of inferred trees whose distance from the corresponding reference trees is less than 0.074. “Bad” are numbers of inferred trees whose distance from the corresponding reference tree is greater than 0.185. Other notations are the same as in Table 9

**Table 12:** Results of the programs on 500 simulated nucleotide alignments

Value	PQ	ME	ML	MP	QP
<*D*>	0.259	0.248	0.218	0.150	0.277
*r*_*DL*_	− 0.12	− 0.15	− 0.31	− 0.22	− 0.15
Perfect	20	28	51	86	10
Good	177	204	253	362	146
Bad	95	85	79	17	108
PQ is better	NA	118	153	58	154
PQ is worse	NA	165	245	341	76
*P*-value	NA	0.006	4·10^−6^	7·10^−50^	3·10^−7^

“Good” are numbers of inferred trees whose distance from the corresponding reference trees is less than 0.1667. “Bad” are numbers of inferred trees whose distance from the corresponding reference tree is greater than 0.3333. Other notations are the same as in Tables 9 and 11

On nucleic acid simulations, MP is the best, even better than ML. Here ME works slightly better than PQ, while QP becomes the worst method.

These results dramatically differ from the results on natural sequences. It means that the used simulation procedures produce alignments that are not realistic and cannot be used for comparison of phylogenetic programs. Probably the natural evolution of biological sequences possesses some properties that are not taken into account by standard algorithms for its computer simulation.

### Long-branch attraction

**Fig. 1:**
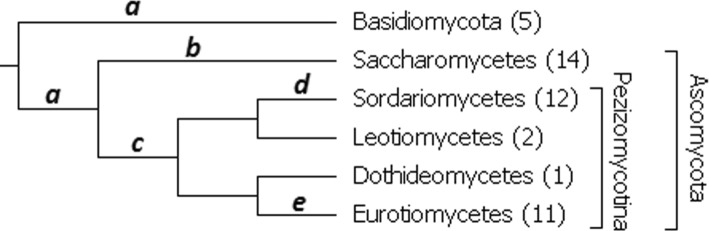
Tree of 45 Fungi. The tree of 45 Fungi labeled with two phyla: Basidiomycota and Ascomycota, subphylum Pezizomycotina and five classes of Ascomycota. Letters a, b, c, d, and e denote branches that must be reconstructed by at least one program for using an orthologous group while investigating long-branch attraction. These branches are: a branch separating two phyla (a), a branch separating Pezizomycotina (c), and three branches separating well-represented classes of Ascomycota (b, d, e)

Long-branch attraction (LBA) often occurs as an artifact in phylogenetic reconstruction [25, 26]. We attempted to investigate the frequency of LBA using our set of fungal alignments when reconstructing phylogeny with the four programs we tested. From 827 alignments of 45 fungal orthologous sequences, we selected alignments satisfying the following condition: each of five branches marked by letters in Fig. 1 was reconstructed by at least one of the four tested programs. Two hundred ninety five such alignments are available in our data.

Next, each of those 295 alignments was restricted to 18 sequences. First, we removed sequences from two poorly represented classes: Leotiomycetes and Dothidiomycetes. Then, in each alignment, we found a sequence among Saccharomycetes that has the maximal mean distance from sequences of Eurotiomycetes. The same was done for sequences from Sordariomycetes. The species set for each of the 295 selected orthologous groups consists of all five Basidiomycota, all 11 Eurotiomycetes, and the two most-rapidly evolving sequences, one from Saccharomycetes and one from Sordariomycetes.

To evaluate the degree of unevenness of the evolution rate in our data, for each of 295 selected protein families we computed the ratio of two values: the first is the average distance from the “fastest” sequence of Saccharomycetes to all sequences from Eurotiomycetes, the second is the average distance between sequences of these two classes. For different protein families, this ratio is proved to be between 1.03 and 3.89, in average 1.18.

**Fig. 2:**
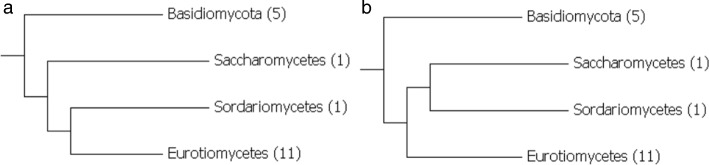
Putative long-branch attraction. **a** The correct tree for 18 fungal species; among Saccharomycetes and Sordariomycetes only species with the most rapidly evolving proteins have been left. **b** The erroneous tree, which can be formed from long-branch attraction

We intended to observe the attraction of branches adjacent to the two lone and rapidly evolving species (Fig. 2). This effect cannot result from an erroneous selection of orthologs, because with the selected 45-species alignments the Pezizomycotina branch was reconstructed.

We reconstructed phylogenies from the restricted alignments with PQ, ME, ML, MP, and QP. For each program, we counted the number of trees containing an erroneous split that separates two lone sequences from others (as in Fig. 2b). Such a split appears in 13 PQ trees, 17 ME trees, 20 ML trees, 20 MP trees, and 18 QP trees.

We repeated the same test, switching Sordariomycetes and Eurotiomycetes so that all Sordariomycetes sequences remained, and the most-rapidly evolving sequences were chosen from Saccharomycetes and Eurotiomycetes. The results of this test are close to those from the first test. An erroneous split appears in 7 PQ trees, 16 ME trees, 13 ML trees, 12 MP trees, and 14 QP trees.

The alignments and trees are available in Additional file 5.

To estimate the statistical significance of comparison of two programs, say A and B, we applied the following procedure. Let *m* be the number of orthologous groups (among the 295 selected) for which A in both described tests does not produce LBA while B at least once in the two tests produces a tree with LBA. Then let *n* be the number of orthologous groups with the opposite situation, namely A produces LBA at least once and B in both tests does not make this error. Now compare *m* and *n* with the sign test.

This procedure shows that PQ outperforms other tested methods with respect to susceptibility to LBA. Namely, with PQ as A and other programs as B, the results of the described procedure (*m*/*n*) are as follows:

- B=ME: *m*/*n*=15/4,*p*=0.01
- B=ML: *m*/*n*=17/2,*p*=3.6·10^−4^
- B=MP: *m*/*n*=20/5,*p*=2·10^−3^
- B=QP: *m*/*n*=13/0,*p*=1.2·10^−4^

## Conclusion

PQ effectively reconstructs phylogenetic trees following a new character-based criterion. Our tests indicate that PQ, at least on alignments of 45 and less relatively short sequences, is more accurate than methods that use the maximum parsimony and maximum likelihood criteria. For sets of 10 or 15 sequences, PQ outperforms the FastME program, which is based on the minimum-evolution criterion. A test on susceptibility to long branch attraction shows that PQ may be the algorithm least susceptible to this problem. PQ, therefore, provides an effective alternative for phylogenetic reconstruction in some situations.

Also, we confirmed the result of G. Gonnet that distance-based methods (in our case FastME) outperform maximum likelihood in accuracy on natural sequences. This result is not supported by simulation studies that suggest an unsatisfactory quality of the existing simulation algorithms.

## Availability and requirements

The datasets supporting the conclusions of this article are included within the article and its additional files.

The described software is available online:

- Project name: PQ
- Project home page: http://mouse.belozersky.msu.ru/software/pq/
- Operating systems: Platform independent
- Programming language: C
- License: GNU GPL

## Additional files

Additional file 1 Organism sets. The MS-Excel file Organisms.xlsx contains lists of organisms from three species sets, with the Uniprot mnemonics that are used in Additional files 2 and 3. (XLSX 23 kb)

Additional file 2 Protein data. The archive Protein-data.tar.gz contains nine folders that each hold data of one data set used in this work. Each folder contains two subfolders called Alignments and Trees. Subfolder Alignments contains sequence alignments in fasta format. Names of the files are Pfam identifiers with additional figures, for example, the file PF00012_3.fasta contains an alignment of the sequences of protein domains from the third orthologous group of Pfam family PF00012. Names of the sequences in alignments are Uniprot organism mnemonics. Subfolder Trees contains five subfolders, PQ, MP, ML, ME, and QP with trees in Newick format reconstructed from the alignments with five methods. Names of the tree files correspond to names of alignment files. The subfolder Trees of folders Metazoa-25, Fungi-45 and Proteobacteria-45 also contains the three species trees used as reference, in Newick format and as PNG images. On the metazoan tree image, all nontrivial branches are labeled with taxon names. On the fungal tree image, branches corresponding to phyla, subphyla, and classes of Pezizomycotina are labeled. On the proteobacterial tree image, branches corresponding to classes are labeled. (TAR 28,930 kb)

Additional file 3 Nucleic acid data. The archive Nucleic-data.tar.gz contains two folders called Fungi and Proteobacteria. Each folder contains two subfolders called Alignments and Trees. Subfolder Alignments contains the alignment of small ribosomal subunit RNA of corresponding organisms and 100 15-sequence subalignments in fasta format. Names of the sequences in alignments are Uniprot organism mnemonics. Subfolder Trees contains five subfolders, PQ, MP, ML, ME, and QP with trees in Newick format reconstructed from the alignments with five methods. Names of the tree files correspond to names of alignment files. Also the subfolder Trees contains the species trees used as reference, in Newick format and as PNG images. On the fungal tree image, branches corresponding to phyla, subphyla, and classes of Pezizomycotina are labeled. On the proteobacterial tree image, branches corresponding to classes are labeled. (TAR 456 kb)

Additional file 4 Simulated data. The archive Simulated-data.tar.gz contains two folders called Alignments and Trees. Folder Alignments contains 500 simulated 15-sequence nucleotide alignments in fasta format. Folder Trees contains six subfolders, PQ, MP, ML, ME, QP and Reference with trees in Newick format reconstructed from the alignments with five methods and with reference trees used for simulations. Names of the tree files correspond to names of alignment files. (TAR 1860 kb)

Additional file 5 Data for testing long branch attraction artifact. The archive LBA.tar.gz contains two folders called Test1 and Test2. Each folder contains two subfolders called Alignments and Trees. Their content are similar to the content of the corresponding files in Additional file 2. Alignments and trees in the folder Test1 each includes sequences from 18 fungal species: five Basidiomycota, 11 Eurotiomycetes, one from Sordariomucetes and one from Saccharomycetes. Alignments and trees in the folder Test2 each includes sequences from 19 fungal species: five Basidiomycota, 12 Sordariomycetes, one from Eurotiomycetes and one from Saccharomycetes. (TAR 1700 kb)

Additional file 6 Scripts. The archive Scripts.tar.gz contains Bash and Python 2.7 scripts used for obtaininh results of the paper and the file ReadMe.txt with their description. (TAR 19 kb)

## Abbreviations

HGT: Horizontal gene transfer
LBA: Long-branch attraction
MP: Maximum parsimony
ML: Maximum likelihood
ME: Minimum evolution
NNI: Nearest neighbor interchange
QP: Quartet puzzle
SPR: Subtree pruning and regrafting
TBR: Tree bisection and reconnection
